# Wild-type MIC distribution and evaluation of epidemiological cut-offs of second-line TB-drugs in susceptible and MDR-TB clinical isolates from Chennai, India

**DOI:** 10.3389/fmicb.2026.1739149

**Published:** 2026-03-11

**Authors:** Azger Dusthackeer, Mahizhaveni Balasubramanian, Sam Ebenezer Rajadas, Christy Rosaline Nirmal, Padmasini Elango, Kannan Thiruvengadam, Shainaba A. Saadhali, Sivakumar Shanmugam

**Affiliations:** 1 Department of Bacteriology, ICMR-National Institute for Research in Tuberculosis, Chennai, India; 2 Department of Statistics, ICMR-National Institute for Research in Tuberculosis, Chennai, India

**Keywords:** drug resistance, epidemiological cut-off values, minimum inhibitory concentration (MIC), *Mycobacterium tuberculosis*, pharmacodynamics, second-line anti-TB drugs

## Abstract

**Introduction:**

The rise of drug-resistant tuberculosis poses a significant challenge in patient management. Epidemiological cut-off values define drug resistance in *Mycobacterium tuberculosis*. In our previous study, we reported deviations from the WHO-recommended epidemiological cut-off values and identified subtherapeutic concentrations of rifampicin in clinical *Mycobacterium tuberculosis* isolates. Building on these findings, the present study systematically evaluated the epidemiological cut-off values and pharmacodynamic profiles of newer and repurposed second-line anti-TB drugs - Bedaquiline, Delamanid, Moxifloxacin, Linezolid, Clofazimine, Levofloxacin, and Pretomanid against the first-line drug-sensitive and isolates that are resistant to Rifampicin and Isoniazid from tuberculosis patients in and around Chennai.

**Methods:**

The Broth microdilution-based Microscopic Observation Drug Susceptibility assay was employed to determine the minimum inhibitory concentration of the drugs against well-characterized wild-type and drug-resistant clinical *Mycobacterium tuberculosis* clinical isolates. The resulting MIC profiles were subsequently analyzed to delineate pharmacodynamic relationships underlying therapeutic efficacy and resistance development.

**Results and discussion:**

Deviations from the World Health Organization–recommended epidemiological cut-off values were observed, with lower thresholds for delamanid and levofloxacin and higher concentrations for clofazimine and bedaquiline. These shifts indicate region-specific susceptibility patterns in *Mycobacterium tuberculosis* that have direct implications for the effective treatment of multidrug-resistant tuberculosis. Inaccurate cut-off values may lead to misclassification of resistance, inappropriate regimen selection, and exposure to suboptimal drug concentrations, thereby compromising treatment efficacy and amplifying the risk of acquired resistance. Concordantly, pharmacodynamic analyses revealed sub-therapeutic exposure ranges for several newer and repurposed anti-TB drugs, underscoring the potential for treatment failure even in strains classified as susceptible. Collectively, these findings highlight the urgent need for regionally calibrated epidemiological cut-off values to optimize drug dosing, improve MDR-TB treatment outcomes, and strengthen resistance surveillance frameworks.

## Introduction

Tuberculosis (TB) remains one of the leading infectious causes of morbidity and mortality worldwide, with an estimated 10.7 million new cases reported annually despite sustained global control efforts. Of these 10.7 million, 4% were Multi-Drug Resistant TB cases. India bears the highest burden of TB globally, accounting for approximately one-quarter of the world’s incident cases, making TB control a major public health priority in the country (Padhi et al., 2024; Singh et al., 2025). According to the Global TB Report 2025, India continues to face a dual challenge of high TB transmission and a growing burden of drug-resistant TB (DR-TB), which threatens progress toward TB elimination targets (World Health Organization, 2025). Effective TB management hinges on timely diagnosis, accurate drug susceptibility testing (DST), and optimized treatment regimens tailored to prevailing resistance patterns.

Drug-susceptible TB (DS-TB) is defined as a disease caused by *Mycobacterium tuberculosis* (Mtb) strains susceptible to first-line anti-TB drugs, whereas multidrug-resistant TB (MDR-TB) is characterized by resistance to at least isoniazid (INH) and rifampicin (RMP), the two most potent first-line agents. More advanced resistance patterns include pre-extensively drug-resistant TB and extensively drug-resistant TB (XDR-TB), which involve additional resistance to fluoroquinolones and/or newer Group A drugs (Viney et al., 2021; Dheda et al., 2024). Clinically, TB diagnosis relies on a combination of microbiological confirmation (smear microscopy, culture, and nucleic acid amplification tests such as GeneXpert MTB/RIF), radiological findings, and clinical assessment, with rapid molecular diagnostics now forming the backbone of early MDR-TB detection under national and WHO-recommended algorithms (Xi et al., 2022; Dheda et al., 2024).

For DS-TB, standardized first-line treatment regimens comprising INH, RMP, ethambutol, and pyrazinamide are highly effective, achieving treatment success rates exceeding 85% under programmatic conditions. In contrast, MDR-TB requires prolonged and complex treatment using second-line and newer anti-TB drugs (Central TB Division, 2024). Current WHO and Indian national guidelines recommend all-oral, shorter or longer MDR-TB regimens incorporating fluoroquinolones [levofloxacin (LFX) or moxifloxacin (MFX)], bedaquiline (BDQ), linezolid (LZD), clofazimine (CFX), delamanid, and pretomanid (PA824), depending on drug susceptibility patterns. Accurate classification of resistance is therefore critical, as misclassification can result in inappropriate regimen selection, reduced treatment efficacy, and amplification of resistance (Mirzayev et al., 2021).

The designation of drug resistance in Mtb relies on epidemiological cut-off values (ECOFFs), defined as the lowest drug concentration inhibiting at least 95% of phenotypically wild-type strains. ECOFFs are foundational for DST interpretation, surveillance of resistance trends, and the rational design of treatment regimens (Werngren et al., 2012; CRyPTIC Consortium, 2023; Centre for Disease Control and Prevention, 2024). However, accumulating evidence suggests that ECOFFs may not be universally applicable across geographic regions due to the evolutionary adaptation of Mtb under diverse drug pressures. Shifts in MIC distributions within wild-type populations can result in discordance between laboratory susceptibility results and clinical drug exposure, thereby undermining treatment outcomes (Schon et al., 2009; Juréen et al., 2010; Werngren et al., 2012).

The evolution of Mtb, driven by genetic mutations and probable selective pressure from widespread drug use, is among the important reasons behind the observed significant shifts in drug susceptibility profiles. These changes are not confined to resistant strains but also affect wild-type populations of Mtb (Allué-Guardia et al., 2021). As wild-type strains adapt to varying environmental conditions and drug exposures, their MICs can alter, potentially rendering the current ECOFFs obsolete (Jones et al., 2022; Nimmo et al., 2022). Understanding the dynamics of ECOFFs in wild-type Mtb is crucial for several reasons. Firstly, it helps in the accurate diagnosis of drug resistance, ensuring that patients receive the most effective treatments promptly. Secondly, it informs the revision of treatment guidelines, which is essential for maintaining the effectiveness of the TB control programs. Thirdly, it aids in developing new drugs and treatment regimens by providing a clear picture of the drug susceptibility landscape. In our previous investigation, we observed a deviation in the ECOFFs of INH from the World Health Organization (WHO) recommended ECOFF and the sub-therapeutic serum concentration of RMP in certain patients (Dusthackeer et al., 2020). This has raised concerns about the hyper-reporting of INH-resistant TB cases. The outcome of the study inspired us to study the MIC distribution of second-line anti-TB drugs, whose ECOFFs are vital to designate the isolate as XDR. We further proceeded to evaluate the ECOFFs of second-line anti-TB drugs - BDQ, DLM, MFX, LZD, CLF, LFX, and PA824. The recent introduction of newer and repurposed drugs for MDR-TB treatment in India necessitates understanding the pathogen’s response to these drugs to design effective therapeutic regimens. By contextualizing MIC variability within regional epidemiology, this study aims to inform optimized DST interpretation, improve regimen design, and strengthen MDR-TB treatment and resistance surveillance strategies in high-burden settings.

## Materials and methods

### Bacterial isolates

Two sets of Mtb clinical isolates, consisting of 110 wild-type strains and the other included 110 strains resistant to first-line anti-TB drugs, were used in this study. These archived clinical isolates were previously collected from presumptive pulmonary TB patients visiting public healthcare facilities in and around Chennai, Tamil Nadu, India, before the initiation of anti-TB treatment in case of drug-susceptible isolates. Sample collection was done over a period of 3 years between 2020 and 2023. Drug susceptibility was originally tested using the Mycobacterial Growth Indicator Tube (MGIT) method for the drugs tested in this study. These cultures were retrieved and revived on Lowenstein-Jensen (LJ) media with approval from the Institutional Ethics Committee of ICMR-National Institute of Research in Tuberculosis. Repeated sub-culturing was avoided to prevent a possible shift in the physiological and phenotypical characteristics of the isolates. Patient identifiers were removed, and no further contact was established with patients; only the laboratory data about these cultures were used. It was ensured that these cultures had not undergone more than two subcultures.

### MIC determination

Broth-Microdilution-based Microscopic-Observation Drug- Susceptibility (BMD) assay in Middlebrook 7H9 broth supplemented with Oleic acid, Dextrose, and Catalase (OADC) (Becton, Dickinson, USA), was carried out following the method as described by the CRyPTIC Consortium, 2022, to determine the MIC for each drug against Mtb clinical isolates, including both wild-type and MDR strains (Schon et al., 2009; Juréen et al., 2010; Makane et al., 2019; Dusthackeer et al., 2020; CRyPTIC Consortium, 2022). In brief, a mid-log phase Mtb clinical culture (20–25 days old) with a homogenous suspension was prepared from LJ slants. From this, a 0.5 McFarland standard suspension was prepared and diluted 100-fold (100 μl of suspension is added to 9.9 mL of 7H9 broth) using enriched Middlebrook 7H9 medium and was used as inoculum. In a 96-well microtiter plate, 100 μl of inoculum (∼1.5 × 10^5^ CFU/mL) is added to each well uniformly. These were then treated with a range of BDQ, DLM, MFX, LZD, CFZ, LFX, and PA824 concentrations (Table 1). The stocks of these drugs were prepared using the solvents as suggested by the manufacturer, and the dilutions were prepared using Middlebrook 7H9 medium supplemented with OADC. Culture control and DMSO control were included. After incubating the plates for 14 days at 37 °C, Mtb growth was assessed under an inverted light microscope, identifying serpentine cords in the control wells initially. The minimum concentration that fully inhibited Mtb growth was recorded as the MIC. All experiments were conducted in duplicate and repeated in 10% of the cultures to ensure reproducibility. The Mtb H37Rv laboratory strain served as an internal culture control.

**TABLE 1:** Minimum inhibitory concentration (MIC) test concentration range.

Drug name	Concentration range (μg/mL)	
	Sensitive Mtb isolates	First line DR-TB isolates
BDQ	0.015, 0.03, 0.06, 0.125, 0.25, 0.5, 1, 2, 4, 8	0.015, 0.03, 0.06, 0.125, 0.25, 0.5, 1, 2, 4, 8, 16
DEL	0.0008, 0.0017, 0.003, 0.006, 0.013, 0.0137, 0.0275, 0.055, 0.11, 0.22, 0.44	0.0008, 0.003, 0.006, 0.008, 0.0137, 0.015, 0.0275, 0.055, 0.06, 0.11, 0.22, 0.44
MFX	0.015, 0.03, 0.06, 0.125, 0.25, 0.5, 1, 2, 4, 8	0.015, 0.03, 0.06, 0.125, 0.25, 0.5, 1, 2, 4, 8, 16
LZD	0.015, 0.03, 0.06, 0.125, 0.25, 0.5, 1, 2, 4, 8	0.015, 0.03, 0.06, 0.125, 0.25, 0.5, 1, 2, 4, 8, 16
CFX	0.015, 0.03, 0.06, 0.125, 0.25, 0.5, 1, 2, 4, 8	0.015, 0.03, 0.06, 0.125, 0.25, 0.5, 1, 2, 4, 8, 16
LFX	0.015, 0.03, 0.06, 0.125, 0.25, 0.5, 1, 2, 4, 8	0.015, 0.03, 0.06, 0.125, 0.25, 0.5, 1, 2, 4, 8, 16
PA824	0.03, 0.06, 0.125, 0.25, 0.5, 1, 2, 4, 8, 16	0.015, 0.03, 0.06, 0.125, 0.25, 0.5, 1, 2, 4, 8, 16

### Whole genome sequencing (WGS)

The Genomic DNA Clean and Concentrator kit (Zymo Research, Irvine, CA, USA) was used to purify genomic DNA isolated from clinical Mtb isolates using the CTAB (cetyltrimethylammonium bromide) method. Using the Nano DropTM and QubitTM dsDNA test kit method (ThermoFisher Scientific, Waltham, MA, USA), the quality and amount of purified DNA were evaluated. The NEXTERA-XT DNA library preparation kit was used to create sequencing libraries. In short, DNA fragments were subjected to several enzymatic steps for end-repairing and dA-tailing, which is followed by adapter sequence ligation. To create final libraries for paired-end sequencing on a HiSeq X 10 sequencer (Illumina, San Diego, CA, USA), adapter-ligated fragments were cleaned up using SPRI beads and then indexed using limited-cycle PCR. Trimmomatic v0.36 was used to filter reads that were at least 30 bp long (150 bp read length) and had a minimum base quality of 20. Using Kraken v1.0, contamination with other species was ruled out. With bwa v0.7.12 and default settings, reads were mapped to the H37Rv reference genome (NC_000962.3). Picard v2.2.4^1^ and GATK v3.5^2^ were used to rectify mapping at indels. Samtools v1.3.1 was used with default parameters to identify variants. The following criteria were used to select the variants: read depth > 5, mapping quality > 30, base quality > 30, and at least one read mapping in either direction. Heterozygous sites were defined as those with <80% mapped reads, whereas homozygous sites were defined as those supported by >80% of the mapped reads. The variants obtained were referred to and compared to a database of resistance-conferring variants that was compiled from reports of previous studies (Shanmugam et al., 2022). In case of BDQ and CFZ, atpE, Rv0678, pepQ and promoter/regulatory regions upstream of Rv0678; in case of DLM and PA824, ddn, fgd1, fbiA, fbiB, fbiC and cofC; In case of fluoroquinolones, gyrA and gyrB; in case of LZD, rrl (23S rRNA) and rplC were the resistant locus examined.

### Determining the pharmacodynamics values

To study the pharmacodynamics, Cmax/MIC and AUC_24hrs_/MIC for each isolate are calculated. The Cmax and AUC_24hrs_ values for each drug were taken from earlier reported population PK studies retrospectively (Pea et al., 2010; Winter et al., 2013; Park et al., 2015; Abdelwahab et al., 2020; Bhatnagar et al., 2024) and the values are given in Table 2.

**TABLE 2:** Optimal PK levels for anti-TB drugs.

Drugs	Cmax (μg/mL)	AUC_24h_ (μg*h/mL)	References
BDQ	2.1	27.3	Bhatnagar et al., 2024
CLF	0.363	7.33	Abdelwahab et al., 2020
DLM	0.3	5.1	Bhatnagar et al., 2024
LFX	6.6	64.4	Park et al., 2015
MFX	4.7	54.2	Park et al., 2015
PA824	1.13	28.08	Winter et al., 2013
LZD	14.7	196.08	Pea et al., 2010

The Cmax/MIC and AUC_24hrs_/MIC were calculated for each sensitive strain and resistant strain using the MIC values deduced in this study. The results of Cmax/MIC and AUC_24hrs_/MIC were then compared with the cut-off values of AUC_24hrs_/MIC for DLM, LFX, MFX, and LZD (Gumbo et al., 2004; Srivastava et al., 2017; Deshpande et al., 2018; Mallikaarjun et al., 2020; Zheng et al., 2021, 2022). For some drugs, the cut-offs were not available in literature (Cmax/MIC and AUC_24hrs_/MIC cut-off values were not available for BDQ, CFZ, and PA824. Cmax/MIC alone were not available for DLM, MFX and LZD among drug susceptible isolates; for Drug resistant isolates both the cut-offs were not available for all the tested drugs except for AUC_24hrs_/MIC of LZD), and so 10 is used for Cmax/MIC and 100 is used for AUC24/MIC based on earlier literature for the drugs which were devoid of any reported cut-off values specific for each (Hemanth Kumar et al., 2016; Dusthackeer et al., 2020). The cut-off values are given in Table 3.

**TABLE 3:** PD cut-offs.

Drugs	Sensitive		Resistant	
	Cmax/MIC	AUC_24_/MIC	Cmax/MIC	AUC_24_/MIC
BDQ	10	100	10	100
CLF	10	100	10	100
DLM	10	195 (Mallikaarjun et al., 2020)	10	100
LFX	12.2 (Deshpande et al., 2018)	146 (Deshpande et al., 2018)	10	100
MFX	10	56 (Gumbo, 2010)	10	100
PA824	10	100	10	100
LZD	10	119 (Srivastava et al., 2017)	10	125 (Srivastava et al., 2017)

While comparing, the strains were classified as therapeutic if the PD-derived (Cmax/MIC and AUC_24hrs_/MIC) met the cut-off values and classified as sub-therapeutic if it is below the cut-off values.

### Statistical analysis and sample size calculation

Data were recorded in Microsoft Excel and analyzed using STATA version 15.1 (StataCorp, Texas, USA). Frequency distributions and percentages were calculated and presented through bar charts. The distribution of MIC values for wild-type Mtb strains was assessed by determining the 95^th^ percentile of observed MIC values.

The sample size was planned to allow reliable analysis of MIC distributions for key anti-TB drugs, including BDQ, LZD, CFX and LFX in both drug-susceptible and drug-resistant Mtb isolates. Since MIC ranges and critical concentrations vary across drugs, variability was considered in relative terms, assuming an approximate ±50% variation around a reference MIC level to reflect realistic differences across drugs. Based on this assumption, at least 100 isolates were considered necessary to obtain stable MIC distribution estimates. To account for possible culture contamination and data loss, the sample size was increased by 10%. In addition, to ensure adequate representation of both susceptible and resistant strains, including MDR- and XDR-TB, the study included 110 drug-susceptible and 110 drug-resistant isolates, giving a total of 220 isolates.

## Results

**FIGURE 1:**
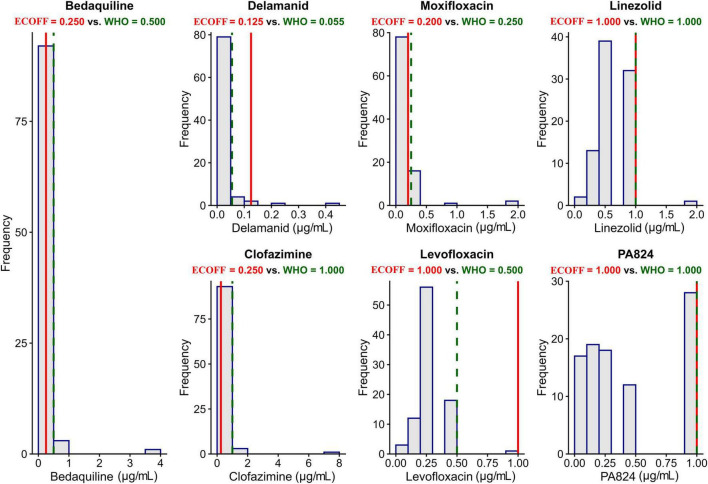
Minimum inhibitory concentration (MIC) distribution and ECOFF of 2nd line anti-TB drugs for wild-type Mtb strains. WHO-recommended ECOFF is shown as a red continuous line, and green dotted lines represent observed ECOFF. Except LZD and PA824, all the other drugs tested have a deviation in ECOFF from WHO WHO-recommended ECOFF.

**FIGURE 2:**
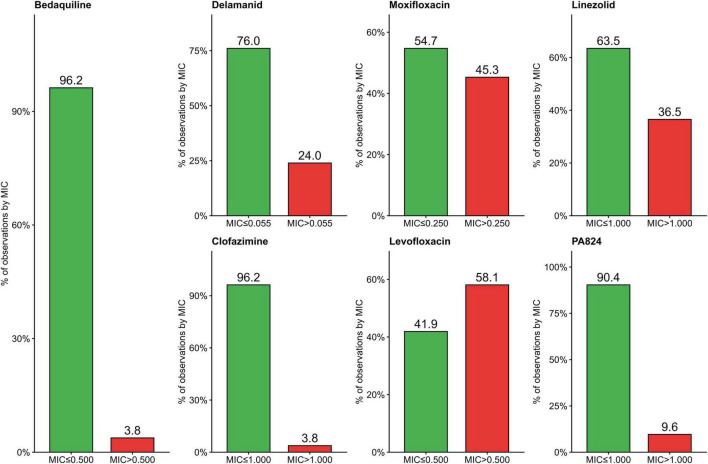
Minimum inhibitory concentration (MIC) distribution of 2nd-line anti-TB drugs in the Mtb clinical strains resistant to 1st-line anti-TB drugs. The percentage of isolates among drug-resistant isolates tested, which were seen above the observed CCs in the study, were 3.8%, 24%, 45.3%, 36.5%, 3.8%, 58.1%, and 9.6% for BDQ, DEL, MFX, LZD, CFZ, LFX, and PA824, respectively.

The MICs for BDQ, DLM, MFX, CFZ, PA824, LZD, and LFX were determined for wild-type isolates and first-line drug-resistant isolates. Deviations from the WHO-determined CCs for BDQ, DLM, MFX, CFZ, and LFX in the wild-type (Figure 1) were seen. In the wild-type strains, there was a one-fold higher difference for BDQ and a two-fold higher difference for CFZ, a one-fold lower difference for DLM and LFX, and a meager difference from 0.25 to 0.20 for MFX was observed when compared to the ECOFF reported by the WHO. Observed ECOFF of LZ and PA824 was in coherence with WHO recommendations. The MIC distribution of 2nd-line anti-TB drugs in the Mtb clinical strains resistant to 1st-line anti-TB drugs was determined (Figure 2).

Whole genome sequencing was performed for 28 wild-type Mtb strains exhibiting MICs above the WHO-defined ECOFFs but less than or equal to the predicted ECOFFs established in this study. The objective was to assess potential resistance to the tested drugs by screening for globally reported resistance-associated mutations, as described previously (Battaglia et al., 2020; Uma Devi, 2022). No known resistance-conferring mutations were identified in any of the analyzed genomes (data not shown). In addition, all sequences were cross-validated against the WHO Catalog (2^nd^ Edition) of mutations in the Mtb complex and their association with drug resistance, the most recent version available, to confirm the absence of documented resistance-associated variants (World Health Organization, 2023). The whole-genome sequencing data have been deposited in the NCBI database, and the corresponding accession numbers are provided in Table 4.

**TABLE 4:** Accession number of strains whose sequences were deposited in NCBI.

S. no	Accession no	S. no	Accession no
1	SAMN45919765	15	SAMN45919751
2	SAMN45919764	16	SAMN45919750
3	SAMN45919763	17	SAMN45919749
4	SAMN45919762	18	SRR31099364
5	SAMN45919761	19	SRR31099119
6	SAMN45919760	20	SRR31099117
7	SAMN45919759	21	SRR31099120
8	SAMN45919758	22	SRR31099118
9	SAMN45919757	23	SRR31099127
10	SAMN45919756	24	SRR31099126
11	SAMN45919755	25	SRR31099116
12	SAMN45919754	26	SRR31099123
13	SAMN45919753	27	SRR31099122
14	SAMN45919752	28	SRR31099121

The most frequently observed MIC ranges for each drug tested among the wild-type Mtb isolates were as follows: BDQ (0.06–0.5 μg/mL), DLM (0.008–0.125 μg/mL), CFZ (0.3–1.0 μg/mL), LZD (0.25–1.0 μg/mL), MFX (0.06–0.25 μg/mL), and LFX (0.125–0.5 μg/mL). The percentage of isolates among drug-resistant isolates tested, which were seen above the observed ECOFFs in the study, was 3.8%, 24%, 45.3%, 36.5%, 3.8%, 58.1%, and 9.6% for BDQ, DEL, MFX, LZD, CFZ, LFX, and PA824, respectively (Figure 2).

### Pharmacodynamic analysis

**FIGURE 3:**
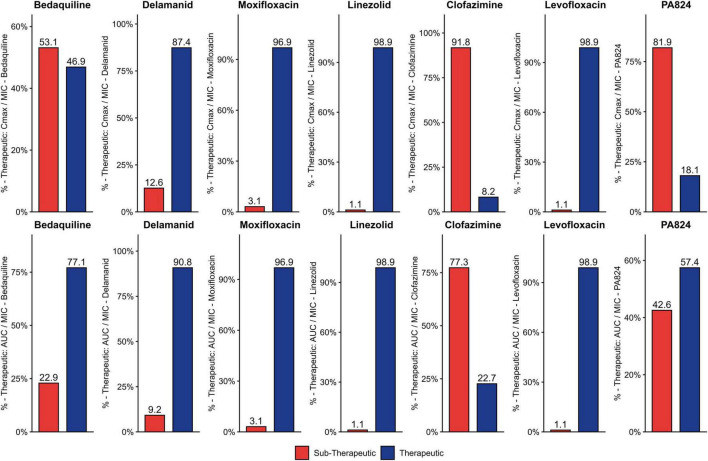
Therapeutic and sub-therapeutic populations among sensitive strains for 2nd line drugs. The percentage of sub-therapeutic and therapeutic populations among the sensitive strains tested for BDQ, DLM, MFX, LFX, CFZ, LZD, and PA824 using Cmax/MIC and AUC_24hrs_/MIC ratios is given. The sub-therapeutic population is given in red, and the therapeutic population is given in dark blue.

**FIGURE 4:**
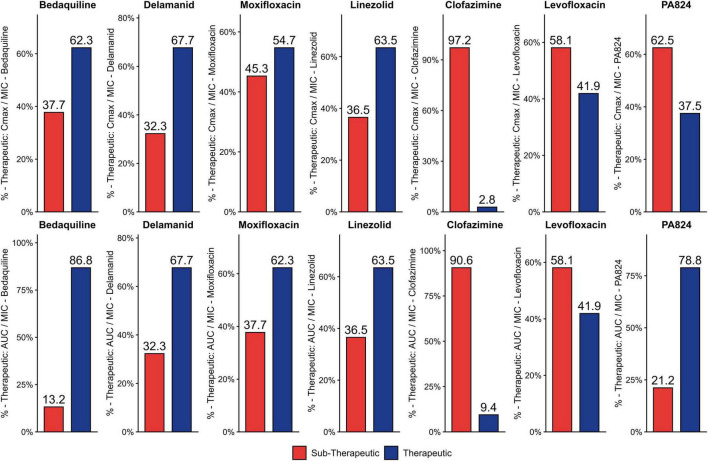
Therapeutic and sub-therapeutic populations among resistant strains for 2nd line drugs. The percentage of sub-therapeutic and therapeutic populations among the resistant strains tested for BDQ, DLM, MFX, LFX, CFZ, LZD, and PA824 using Cmax/MIC and AUC_24hrs_/MIC ratios is given. The sub-therapeutic population is given in red and therapeutic population is given in dark blue.

The PD Values of each strain for the drugs DLM, BDQ, MFX, LZD, LFX, CFZ, and PA824 were compared with the described PD cut-offs, and the percentage of strains that are sub-therapeutic was assessed from Figures 3, 4. Notably, in BDQ, 53.1% of sensitive strains and 37.7% of resistant strains are sub-therapeutic for Cmax/MIC. For CFZ, 91.8% of sensitive strains and 97.2 % of resistant strains are sub-therapeutic for Cmax/MIC. In the case of AUC_24_/MIC for CFZ, 77.3% of sensitive strains and 90.6% of resistant strains are sub-therapeutic. For MFX, 45.3% of resistant strains are sub-therapeutic for Cmax/MIC, and 37.7% of resistant strains are sub-therapeutic for AUC24/MIC. In the case of PA824, 81.9% of sensitive strains and 62.5% of resistant strains are sub-therapeutic for Cmax/MIC. In AUC_24_/MIC, 42.6% of sensitive strains are sub-therapeutic for PA824. In case of DLM, in resistant strains, 32.3% are sub-therapeutic for both Cmax/MIC and AUC_24_/MIC, and most of the sensitive strains are therapeutic. For LZD, most of the strains meet the cut-off values in sensitive strains, and 36.5% of resistant strains were sub-therapeutic for both Cmax/MIC and AUC_24_/MIC. In the case of LFX, most of the sensitive strains fall in the therapeutic range, but 58.1% of resistant strains show sub-therapeutic values for both Cmax/MIC and AUC_24_/MIC.

## Discussion

Programmatic management of MDR-TB in India employs a stepwise DST approach, beginning with Xpert MTB/RIF or Xpert MTB/RIF Ultra for rapid identification of rifampicin resistance, followed by first- and second-line line probe assays to detect resistance to INH, fluoroquinolones, and second-line injectable agents. Phenotypic DST using liquid culture (MGIT 960) is subsequently undertaken for comprehensive susceptibility assessment of second-line and newer drugs, including BDQ, LIN, CFX, and DEL. These combined molecular and phenotypic DST results guide the selection of individualized, all-oral MDR-TB regimens under the National TB Elimination Programme (NTEP), underscoring the clinical consequences of misaligned ECOFF values. Wild-type MIC distributions are critical in defining drug susceptibility, especially while performing liquid DST (Ängeby et al., 2011). However, variability at the strain, non-clinical, and methodological variation has an impact on it and warrants the need for region-wise determination of MIC distribution for each drug (Mouton et al., 2018; Catalán et al., 2022). Consistent with our previous findings on RMP, we observed deviations in the ECOFF values of BDQ and CFZ, which were higher than those established by WHO for the given population. Similar findings have also been reported in other geographical regions (Kaniga et al., 2022). The possibility of hyper-reporting of resistant cases (for BDQ, MFX, and CFZ) and misreporting (for DLM and LFX) arises due to the observed variations in the ECOFFs among the isolates prevalent in this region. This will mislead the treatment providers and further extend to subjecting patients to an overburdened treatment regimen. BDQ and CFZ show strong efficacy with minimal resistance in both sensitive and resistant samples, aligning well with previous reports (Ndjeka et al., 2022; Stadler et al., 2023). However, this result requires in-depth introspection based on the global data. The study was attempted using a broth-microdilution-based microscopic-observation drug-susceptibility method, which is expected to be economical with minimal one-time investment in an inverted microscope and does not require a sophisticated facility with high maintenance charges. The data on ECOFF among strains prevalent in this region underscores the need for regulatory agencies to reassess and optimize ECOFF recommendations for more accurate formulation.

The recent introduction of newer and repurposed drugs in the treatment of MDR-TB in India has made it essential to understand the susceptibility of Mtb against these drugs for effective regimen planning. Given the regional variability in MICs observed in previous studies, such as our exploratory research on first-line anti-TB drugs (Dusthackeer et al., 2020). We continued to investigate the MICs of isolates from retreatment cases. Although previous studies have reported cross-resistance between BDQ and CFZ, this was not observed in the current study, except for two of the four BDQ-resistant isolates, which also exhibited resistance to CFZ based on the observed ECOFF for both drugs. The WHO ECOFF of DLM is slightly higher than the observed ECOFFs of 0.055 μg/mL, and one of the isolates exhibited a MIC of 0.4 μg/mL. In contrast, previous studies, such as those by Stinson et al. (2016), reported two isolates with MICs of 1 and 2 μg/mL among 204 tested, while Pang et al. (2017) identified 3 out of 90 isolates with a significantly high MIC of 32 μg/mL in Shanghai, China (Stinson et al., 2016; Pang et al., 2017). WGS was primarily used to identify drug-resistant mutants among wild-type isolates that exhibited differing ECOFFs compared to the levels recommended by the WHO (World Health Organization, 2023). Beyond canonical resistance mutations, WGS of clinical Mtb isolates has identified mutations associated with antibiotic tolerance and persistence, which enable bacterial survival under drug pressure without conferring high-level resistance (Toloza et al., 2025; Wang et al., 2025). Antibiotic killing assays, such as time–kill kinetics, minimum bactericidal concentration determination, and MDK (minimum duration for killing) assays, can reveal substantial heterogeneity in drug tolerance among clinical isolates, even within phenotypically susceptible populations (Vijay et al., 2021). Variants affecting stress response, redox balance, transcriptional regulation, and efflux control (including relA, prpR, whiB family genes, and Rv0678) have been linked to delayed killing kinetics, increased minimal bactericidal concentrations, and prolonged time to culture conversion. Such tolerance-associated mutations promote survival at borderline or sub-therapeutic drug exposures, increasing the risk of relapse and facilitating stepwise acquisition of stable resistance, thereby limiting the predictive accuracy of MIC and breakpoint-based DST alone (Goossens et al., 2020). However, no such mutations were analyzed in this study, and the underlying causes of sensitivity or resistant patterns require further investigation, involving a larger number of similar cases.

Significantly increased levels of resistance were observed for fluoroquinolones (58.1% for LFX and 45.3% for MFX), likely reflecting the widespread use of these antibiotics within the community. This overuse may be attributed to the availability of these drugs over the counter in India, without the need for a physician’s prescription, which could be a primary factor contributing to the observed rise in resistance. A 5-years surveillance study across 11 countries reported similar findings, with FQ resistance ranging from 33% to 35%. In India, FQ resistance exhibited significant variation, with rates ranging from 17% in Delhi to 70% in Mumbai (Mamatha and Shanthi, 2018; Kaniga et al., 2022). In contrast, a 40.7 % FQ resistance rate was observed in China, which is attributed to hetero resistance. These patterns align with our findings in previously treated MDR-TB patients. Alarmingly, we observed a 19% frequency of MIC at the ECOFF of 0.25 μg/mL for MFX among wild-type isolates, indicating that they might be less susceptible to MFX. However, no similar trend was observed with LFX, which has an ECOFF of 1 μg/mL, with only 2% of wild-type isolates showing MIC at this level. Empiric FQ use for community-acquired respiratory infections may expose individuals with latent or undiagnosed TB to subtherapeutic drug levels, thereby selecting for FQ-resistant Mtb, particularly when exposure precedes TB diagnosis (Devasia et al., 2009). This exposure-driven selection pressure plausibly explains the rightward shift in MIC distributions and lower epidemiological cut-off values observed for levofloxacin and moxifloxacin in the present study.

Linezolid is an essential component of the recently introduced regimen for MDR-TB treatment in India and globally. In this study, among resistant strains, LZD resistance was detected in 36.5% of isolates from patients in and around Chennai, with 25% of these isolates also exhibiting FQ resistance. These findings are consistent with resistance patterns reported in other regions of India, including Mumbai and North India (Vengurlekar et al., 2023). Notably, this study also found resistance in patients with no prior exposure to LZD. A meta-analysis by Azimi et al. (2022) based on data from China and Turkey reported a lower LZD resistance rate of 4.25% among studies conducted from 2000 to 2021 (Azimi et al., 2022). Of particular concern, 24% exhibited MICs approaching the ECOFF of 1 μg/mL in wild-type isolates with no history of TB treatment. These trends suggest that while LZD was introduced in the late 2000s, resistance has been gradually increasing, in both southern and northern India, among the MDR-TB isolates. This highlights the urgent need for rapid screening methods to detect LZD resistance in patients undergoing treatment, ensuring better outcomes while limiting the over-the-counter use of the drug for other infections.

Levofloxacin, LZD, and MFX are of concern due to higher MIC values among MDR-TB isolates. This study emphasizes the substantial need for continuous evaluation of drug efficacy and substantiates the rampant use of these wonder drugs for other infections. The observed variations in MIC values and resistance levels underscore the dynamic nature of microbial resistance, highlighting the possibility of cross-resistance and the significance of adaptive approaches in TB treatment to ensure that medications remain effective over time. Strong advocacy is needed to preserve fluoroquinolones and oxazolidinones for the treatment of TB.

The MIC data in this study are crucial for predicting treatment efficacy, as they help to assess the PD of the regimen in use, assuming drug levels are also measured. In the present study, drug levels for these patients were not measured. Therefore, PD was inferred using literature-derived data, specifically Cmax and AUC for deriving Cmax/MIC and AUC/MIC ratios. Despite the limited availability of established cut-off levels for adequate PD, the PD indices presented interesting insights. The newly introduced drugs, BDQ (53.1%), PA824 (81.9%), and CFZ (91.8%), showed increased (>50%) sub-therapeutic levels in terms of the Cmax/MIC ratio. This suggests that the peak serum concentrations of these drugs are insufficient to effectively eliminate the bacillary population. The AUC/MIC ratio for BDQ, DLM, MFX, LZD, and PA824 was within adequate levels (>50%) in both sensitive and resistant isolates. For concentration-dependent agents such as fluoroquinolones and BDQ, clinical efficacy is driven by achieving critical PK–PD targets, particularly AUC/MIC and Cmax/MIC, which may not be met when Mtb isolates exhibit MICs close to established DST resistance breakpoints. Evidence indicates that such “borderline susceptible” isolates–although categorized as susceptible by current cut-offs–are associated with delayed culture conversion, relapse, and on-treatment emergence of higher-level resistance, reflecting systematic limitations of breakpoint-based DST (Colangeli et al., 2018). In this context, the rightward MIC shifts observed in our study underscore how fixed ECOFF values may inadequately capture clinically relevant resistance, especially for key MDR-TB drugs such as FQ and BDQ. For the CFZ, the ratio is sub-therapeutic, exceeding 75% in both sensitive and resistant isolates, suggesting the need for optimization of dosing frequency. Reports suggest that similar recommendations for BDQ and CFZ (Alffenaar et al., 2015; Cholo et al., 2017) have led to poor early bactericidal activity and suboptimal PK/PD outcomes. This suggests that challenges remain in achieving optimal therapeutic levels for better treatment outcomes. Sub-therapeutic ratios of BDQ and CFZ are clinically significant since they promote resistance and may increase cardiac risk due to QT prolongation (Brust et al., 2021).

The observed regional deviations in ECOFF and sub-therapeutic PD indices emphasize the need for periodic, region-specific re-evaluation of DST breakpoints to prevent misclassification of resistance and inappropriate treatment adjustments. Establishing a centralized MIC and PK/PD surveillance frame work under the Indian National TB Elimination Program would further support evidence-based regimen design and help preserve the long-term efficacy of key anti-TB agents such as BDQ and LZD.

### Limitations

Funding constraints limited the application of whole-genome sequencing across all isolates, and drug-specific PD targets (Cmax/MIC and AUC/MIC) for newer anti-TB agents remain incompletely defined, restricting precise PK–PD interpretation. PK analyses were based on population-level estimates rather than individual patient measurements. Given the limited number of confirmed wild-type isolates per drug and the small number of high-MIC observations, wild-type MIC thresholds were estimated using the 95^th^ percentile; under these conditions, estimation of higher percentiles or application of interval regression may be unstable. Although large consortia datasets (e.g., CRyPTIC) permit more advanced modeling, such approaches are not directly transferable to the present dataset, and our approach should therefore be interpreted cautiously.

## Conclusion

The findings of this study highlight deviations in the MIC distribution of second-line anti-TB drugs compared to the WHO-recommended ECOFF. These discrepancies, particularly for BDQ and CFZ, suggest the potential for misreporting of drug-resistant TB cases that may lead to unnecessary alterations in treatment regimens. Our results emphasize the importance of region-specific MIC assessments and the need for re-evaluation of ECOFF values to ensure accurate diagnosis and effective treatment of TB. Adjusting the ECOFF based on local strain variations will contribute to better TB management and prevent the overuse of potent drugs, preserving their efficacy for future treatment strategies. To optimize treatment outcomes and prevent relapse, it is crucial to reassess the newer drugs in terms of their efficacy, ensuring they reach appropriate PD levels. Furthermore, given the inherent funding constraints of the present study, further large-scale, longitudinal investigations integrating MIC profiling with PD and antibiotic tolerance assays are warranted to comprehensively define region-specific susceptibility patterns and inform national MDR-TB treatment and resistance surveillance strategies.

## Data Availability

The datasets presented in this study can be found in online repositories. The names of the repository/repositories and accession number(s) can be found in the article/supplementary material.

## Footnotes

1
http://broadinstitute.github.io/picard/

2
https://gatk.broadinstitute.org/hc/en-us
